# Brainstem Abnormalities in Myalgic Encephalomyelitis/Chronic Fatigue Syndrome: A Scoping Review and Evaluation of Magnetic Resonance Imaging Findings

**DOI:** 10.3389/fneur.2021.769511

**Published:** 2021-12-17

**Authors:** Todd Nelson, Lan-Xin Zhang, Hui Guo, Luis Nacul, Xiaowei Song

**Affiliations:** ^1^Evaluation and Research, Surrey Memorial Hospital, Fraser Health, Surrey, BC, Canada; ^2^Biomedical Physiology and Kinesiology, Simon Fraser University, Burnaby, BC, Canada; ^3^Department of Human Biology, Physiology, University of Toronto, Toronto, ON, Canada; ^4^Department of Diagnostic Imaging, Tianjin Medical University General Hospital, Tianjin, China; ^5^Complex Chronic Diseases Program, BC Women's Hospital and Health Centre, Vancouver, BC, Canada; ^6^Department of Clinical Research, Faculty of Infectious and Tropical Diseases, London School of Hygiene and Tropical Medicine, London, United Kingdom

**Keywords:** myalgic encephalomyelitis/chronic fatigue syndrome (ME/CFS), brainstem, brain structure and function, brainstem dysfunction, symptoms and expressions, pathologies and mechanisms, magnetic resonance imaging (MRI), neuroinflammation

## Abstract

**Background:** Myalgic Encephalomyelitis/Chronic Fatigue Syndrome (ME/CFS) is a multisystem medical condition with heterogeneous symptom expression. Currently, there is no effective cure or treatment for the standard care of patients. A variety of ME/CFS symptoms can be linked to the vital life functions of the brainstem, the lower extension of the brain best known as the hub relaying information back and forth between the cerebral cortex and various parts of the body.

**Objective/Methods:** Over the past decade, Magnetic Resonance Imaging (MRI) studies have emerged to understand ME/CFS with interesting findings, but there has lacked a synthesized evaluation of what has been found thus far regarding the involvement of the brainstem. We conducted this study to review and evaluate the recent MRI findings *via* a literature search of the MEDLINE database, from which 11 studies met the eligibility criteria.

**Findings:** Data showed that MRI studies frequently reported structural changes in the white and gray matter. Abnormalities of the functional connectivity within the brainstem and with other brain regions have also been found. The studies have suggested possible mechanisms including astrocyte dysfunction, cerebral perfusion impairment, impaired nerve conduction, and neuroinflammation involving the brainstem, which may at least partially explain a substantial portion of the ME/CFS symptoms and their heterogeneous presentations in individual patients.

**Conclusions:** This review draws research attention to the role of the brainstem in ME/CFS, helping enlighten future work to uncover the pathologies and mechanisms of this complex medical condition, for improved management and patient care.

## Introduction

### Myalgic Encephalomyelitis/Chronic Fatigue Syndrome

Myalgic Encephalomyelitis, also known as Chronic Fatigue Syndrome (ME/CFS), is a multisystem medical condition affecting the central nervous, cardiovascular, and immune system functions (1). It is estimated that 17 million people worldwide (2, 3) including over 580,000 Canadians (4) are currently living with ME/CFS, with 25% of that population being either housebound or bedbound (5). ME/CFS is characterized by post-exertional malaise, i.e., a worsening of symptoms following minimal mental or physical exertions, and sensory overload with symptoms lasting for up to several months. Other common symptoms can include autonomic dysfunction, e.g., orthostatic intolerance and postural tachycardia problems (6); cognitive deficits including impairments in attention and working memory (7, 8), and substantial reductions in activity engagement (8). ME/CFS is predominant in females and typically has an onset following an infection, environmental toxin exposure, or traumatic accidents. Due to the absence of diagnostic biomarkers and the heterogeneous disease expression among individual patients, diagnosis of ME/CFS involves the careful consideration of other diseases and conditions with similar symptoms (1, 9). The National Institute for Health and Care Excellence (NICE) guidelines on ME/CFS (10) confirm the non-existence of curative treatments for ME/CFS. There are no Food and Drug Administration or Health Canada approved treatments or cures, presumably owing to its unknown pathologies and mechanisms.

### Brainstem

The brainstem (or brain stem) is a compact brain structure that lies between the cerebrum and the cervical spinal cord. It consists of three components: the midbrain, pons, and the medulla; consisting of both gray matter (GM) and white matter (WM). The brainstem has connections with numerous other brain structures, including the prefrontal cortex, hypothalamus, cerebellum, hippocampus, and sensorimotor cortex and performs conduit, cranial nerve, and integrative functions that are prerequisite for living (11, 12). The conduit function involves the passage for all WM tracts that connect the brain and the body, e.g., the corticospinal tract (delivering motor output from the brain), spinothalamic tract and dorsal column-medial lemniscus (providing sensory input to the brain), and the autonomic nervous system sympathetic and parasympathetic tracts (13). The brainstem also contains the cranial nuclei for cranial nerves 3-12, bringing in sensory input like facial sensation and delivering both voluntary and involuntary motor output (14). The vasomotor and respiratory centers of the medulla and pons (part of the reticular formation) integrate the regulation of heart rate, blood pressure, and breathing (15, 16). The substantia nigra and ventral tegmental area of the midbrain execute dopaminergic control of motivation, concentration, and learning (17, 18). The reticular activating system (RAS) nuclei of the brainstem are fundamental for regulating the sleep/wakefulness cycles and consciousness (19, 20).

Many symptoms shown in patients with ME/CFS may be associated with brainstem dysfunctions of different extents. For example, sleep disorders have been linked to impaired brainstem reticular formation especially the RAS nuclei (3). Dysregulation of sleep/wakefulness cycles can cause decreased consciousness, leading to impaired cognitive function (11). Also, autonomic dysfunctions such as inappropriate adjustments of the heart rate to exertion have been linked to damaged dorsal motor nucleus of the vagus nerve (cranial nerve ten) that innervates the brainstem vasomotor center (21). Furthermore, widespread neuroinflammation as seen in ME/CFS especially post exertion (22) has been related to the role the vagus nerve has in triggering neuroinflammation (21, 23). In addition, altered dopaminergic function can arise from abnormal dopaminergic brainstem areas such as the substantia nigra and the ventral tegmental nuclei (24). Finally, several cardiac and respiratory symptoms such as orthostatic intolerance and abnormal breathing (8) can involve the brainstem, specifically the vasomotor, cardiac, and respiratory centers of the medulla and pons. Taken together, the associations between the typical ME/CFS symptoms and the brainstem functions indicate that the brainstem potentially plays a significant role in ME/CFS pathomechanism.

### Magnetic Resonance Imaging on ME/CFS Patients

Brain magnetic resonance imaging (MRI) has been used in better understanding ME/CFS (7, 22, 25, 26). MRI uses a strong magnetic field to align the protons in the tissues and a radiofrequency pulse to make the selective protons spin out of equilibrium such that when the radiofrequency is removed, the protons realign to the magnetic field. The time it takes for realignment and the energy released result in differing hyperintensities (i.e., brightness) creating imaging contrast between tissue types including WM, GM, and cerebrospinal fluid (CSF). Compared to other common neuroimaging methods, MRI is non-invasive without needing radiation or radioactive/iodinated tracers. These features, together with the relatively high resolution, make MRI preferable for both clinical diagnosis and research.

MRI techniques, such as T1-weighted (T1W) imaging and T2-weighted (T2W) imaging, are commonly used to reveal brain anatomy, parenchymal lesions and other structural changes in WM volume, GM volume, and myelination (27). Diffusion Tensor Imaging (DTI) has been used to capture the rate of water diffusion between cells to create the fiber trajectory map representing WM integrity based on parameters such as the diffusivity and fractional anisotropy (28). In addition, functional MRI (fMRI) can be used to study functional brain response to stimuli or functional connectivity (FC), through detecting regional blood flow changes (e.g., perfusion-weighted Arterial Spin Labeling-ASL) or coupled with neuron activity induced oxygen consumption (e.g., blood oxygen level dependent BOLD echo planner imaging-EPI), either during a resting phase (in absence of implicit brain input/output) or during a task (visual, auditorial, motor, cognitive, etc.). Magnetic Resonance Spectroscopy (MRS) or MRS Imaging (MRSI) can be used to reveal the concentration of the major brain metabolites (e.g., N-acetyl aspartate, Creatinine, Choline), based upon which the brain tissue temperatures may be measured (22, 29).

### Purpose of the Study

Despite a lacking consensus on the pathologies and mechanisms of ME/CFS, MRI studies have started to show the involvement of the brainstem. Emerging studies have reported global and regional GM and WM volume reductions in the patients, while inconsistency exists especially regarding brainstem structural changes (30). Similarly, studies have suggested that ME/CFS patients may recruit additional brain regions or increase brain activities in dealing with the symptoms, although inconsistencies remain (30). Changes in brain metabolites (22, 31), BOLD signal complexity (32, 33), and functional connectivity (33) have also been reported.

Considering the vital functions of the brainstem and the associations of these functions with the variety of ME/CFS symptoms, there presents a need of a review and evaluation of the MRI findings concerning the involvement of the brainstem's role in ME/CFS. This has motivated our research aiming to 1) integrate the relevant MRI findings of the research field, and 2) derive a synthesized evaluation of the brainstem's role in ME/CFS based on the findings. The study is intended to provide new insights to advancing the research line, informing future solutions for improved clinical management and patient care of the complex medical condition. The work is also relevant with addressing the challenges of the ongoing pandemic, given that ME/CFS has become one of the long-term consequences of COVID-19 (34).

## Methods

This scoping review followed the Preferred Reporting Items for Systematic Reviews and Meta-Analyses extension for Scoping Reviews (PRISMA-SR) checklist (35).

### Eligibility Criteria

**Figure 1:**
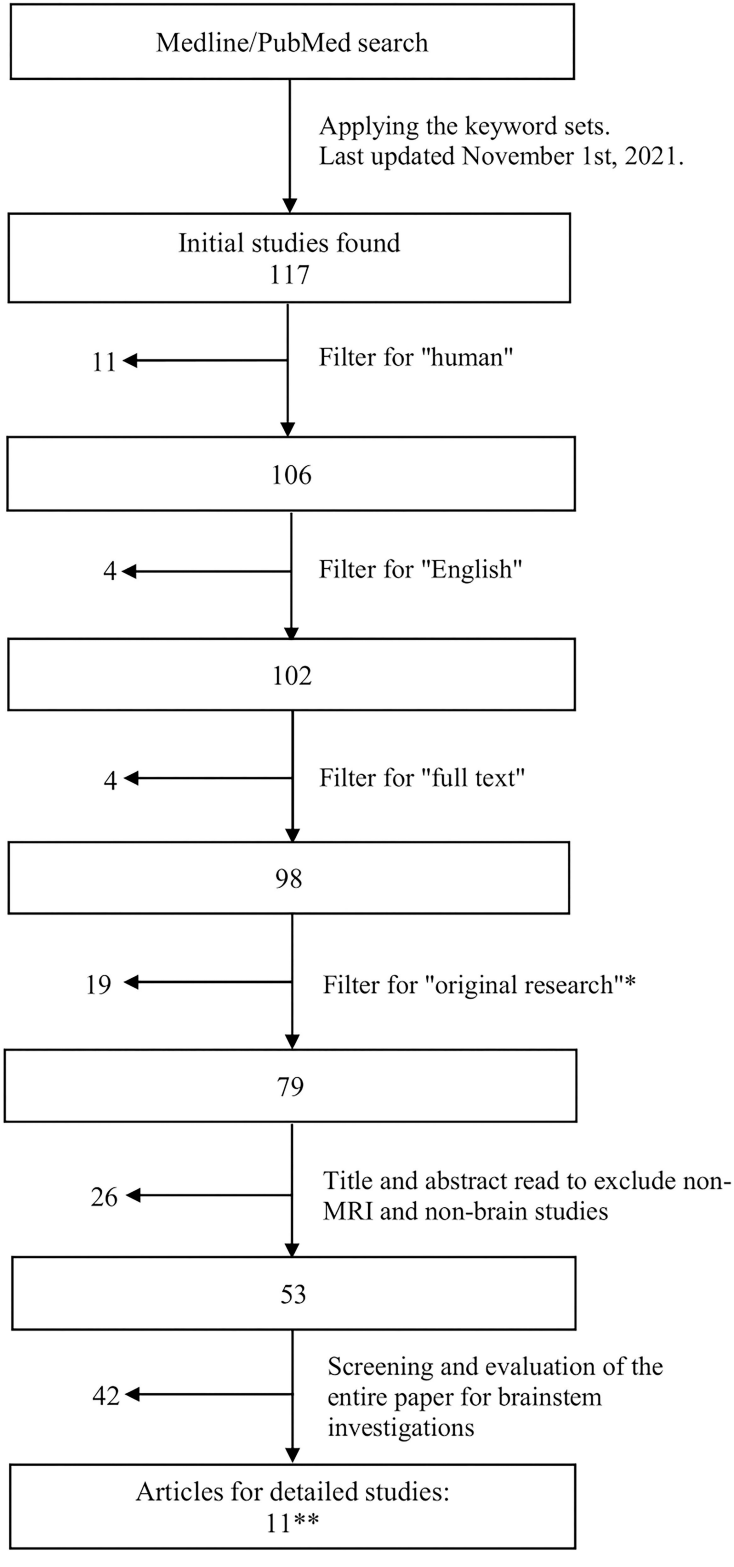
Flowchart showing the literature search and article retrieval process. *Included papers that had original findings, including case reports. **The search revealed a final set of 11 original research studies, none of which were case reports.

To be included in the evaluation, publications must investigate the brainstem in relation to ME/CFS, use MRI techniques, be original research, be conducted on human participants and published in English (as English is the preferred language of this review). Journal articles were included in the search if they were published before November 1st, 2021 (Figure 1).

Papers that only mentioned the brainstem in ME/CFS but did not involve the investigation of the brainstem's role within the study were excluded. Studies that did not fit into the conceptual framework, i.e., the scope and scale of the present review, were filtered out (Figure 1).

### Search Terms

The literature review was conducted by two researchers (TN and LXZ), applying the MEDLINE (the National Library of Medicine's premier bibliographic database that contains 27 million+ references of 5,200 journals in life sciences especially biomedicine) through PubMed search of targeted publications (last updated on November 1st, 2021). Articles were retrieved through subscriptions provided by Simon Fraser University and the University of Toronto.

The initial search was conducted using three sets of keywords: Set 1: “ME^*^CFS” or “Myalgic Encephalomyelitis” or “Chronic Fatigue Syndrome”; Set 2: “MRI” or “Multimodal MRI” or “Magnetic Resonance Imaging” or “fMRI” or “functional Magnetic Resonance Imaging” or “functional MRI” or “MRSI” or “MRS imaging” or “Magnetic Resonance Spectroscopic Imaging” or “MRS” or “Magnetic Resonance Spectroscopy” or “MR Spectroscopy” or “Proton Density Weighted Imaging” or “DWI” or “Diffusion Weighted Imaging” or “DTI” or “Diffusion Tensor Imaging” or “EPI” or “Echo Planar Imaging” or “PWI” or “Perfusion Weighted Imaging” or “ASL” or “Arterial Spin Labeling” or “MRA” or “Magnetic Resonance Angiography” or “MRV” or “Magnetic Resonance Venography” or “Perfusion MRI” or “Perfusion Weighted Imaging” or “PWI” or “anatomical MRI”; and Set 3: “human” or “brain” or “human brain.” This initial search retrieved 117 articles, as shown in Figure 1.

### Selection of Sources of Evidence

Filters were used to select the publication from the initial set of articles (Figure 1). These included “Humans” (to include only human subjects), “English” (to include only English text), and “Full text” (to include only full text). To include only original research (including case reports), the database filters: “Systematic Review,” “Review,” and “Meta-Analysis” were employed. Abstract reading was also done to ensure removal of non-original research articles.

The remaining subset of articles after the filtering steps was subjected to further evaluation in detail. First, a title and abstract read of each paper was conducted to remove articles that were not brain MRI studies on ME/CFS. This was followed by a full text screening to exclude any entries that did not include the brainstem or its components. Thereafter, articles that contained “brainstem, brain stem, pons, pontine, midbrain, medulla” were identified and studied further with full text reading.

These processing steps yielded a final set of 11 articles, all of which were original research publications (rather than case studies or reviews), as presented below (Figure 1 and Table 1). Any disagreements or inconsistencies between TN and LXZ during processing and selection were resolved with additional researchers (e.g., HG and XS).

**Table 1:** MRI studies investigating the role of the brainstem in myalgic encephalomyelitis/chronic fatigue syndrome (ME/CFS).

**References**	**Sample (N)**	**Mean age ± SD**	**Design**	**Structural MRI**	**Functional MRI**	**Main findings**
Barnden et al. (36)*	ME/CFS (25) M: F = 6:19 HC (25) M: F = 6:19	ME/CFS = 31.7 ± 8.8 HC = 33.7 ± 10.3	Cross-sectional case-controlled study	1.5T; T1W imaging spin-echo; T2W imaging spin-echo; 3D spoiled-GRE	None	(1) No regional or global group mean differences in T1W or T2W signal levels, or gray matter (GM) or white matter (WM) volumes (2) Group differences in correlations between structural (e.g., brainstem GM volume; pons, prefrontal cerebellar vermis, and hypothalamus WM volumes, brainstem and cerebellum T1W signal level—suggestive of myelination level) and physiological measures [e.g., seated pulse pressure (PP), diastolic blood pressure (BP), and asleep heart rate (HR)] in multiple brain regions in ME/CFS compared to HC (3) Midbrain WM volume decreased with increasing illness duration in ME/CFS
Barnden et al. (37)*	ME/CFS (25) M: F = 6:19* HC (25). M: F = 6:19*	ME/CFS = 32* HC = 32.8*	Cross-sectional case-controlled study; voxel-based analysis	1.5T; T1W imaging spin-echo; T2W imaging spin-echo; 3D spoiled-GRE	None	(1) Midbrain WM volume decreased with increasing illness duration in ME/CFS (2) T1W signal level increased (suggestive of increased myelination) in ventrolateral thalamus, internal capsule, and prefrontal with increasing ME/CFS symptom severity, presumably in compensation of the midbrain impairment (3) T2W signal level increased (suggestive of decreased regional blood volume) with increasing illness duration in right middle temporal WM in ME/CFS compared to HC
Barnden et al. (38)*	ME/CFS (25) M: F = 6:19* HC (25) M: F = 6:19*	ME/CFS = 32* HC = 32.8*	Cross-sectional case-controlled study	1.5T; T1W imaging spin-echo; T2W imaging spin-echo; 3D spoiled-GRE	None	(1) No regional group differences in T1W or T2W signal levels, or GM or WM volumes in the abnormally correlated brain areas (2) Opposite correlation patterns between multiple MRI measures in focal brainstem structures (e.g., medulla T2W signal level; pons WM volume; hypothalamus T1W signal level and WM volume; cuneiform nucleus and ventral tegmental T1W signal level) and several physiological measures (e.g., erect PP and BP; reclined systolic BP and HR) in ME/CFS compared to HC
Finkelmeyer et al. (39)	ME/CFS (42) M: F = 10:32 HC (30) M: F = 9:19	ME/CFS = 45.6 ± 11.7 HC = 48.4 ± 11.3	Cross-sectional study; whole-brain and voxel-wise GM and WM volume analysis	3.0T; T1W imaging MPRAGE	None	(1) Reduced group mean WM volume in midbrain, pons, internal and external capsules, prefrontal, inferior frontal, and temporal lobe in ME/CFS compared to HC (2) Increased group mean GM volume in parts of the inferior frontal, occipital and temporal lobes, putamen, thalamus, amygdala, and hippocampus in ME/CFS compared to HC (3) Reduced total intracranial volume (TIV), increased TIV-adjusted global GM volume, and decreased TIV-adjusted global WM volume in ME/CFS compared to HC (4) Negative associations between symptom severity and TIV, TIV-unadjusted GM, and cerebrospinal fluid volume in ME, not in HC
Barnden et al. (40)**	ME/CFS (43) M: F = N/A** HC (27) M: F = N/A**	Not available**	Cross-sectional study	3.0T; T1W imaging spin-echo; T2W imaging spin-echo	None	(1) Decreased T1W signal levels (suggestive of decreased myelination) in brainstem (ventral tegmental, pontine nuclei, medulla regions), and increased T1W signal levels in cortical WM regions (premotor and sensorimotor cortices) in ME/CFS compared to HC (2) In both ME/CFS and HC, there was a negative correlation between the T1W signal levels in the sensorimotor cortex (high myelination) and brainstem (low myelination)
Boissoneault et al. (41)	ME/CFS (19) M: F = 0:19 HC (15) M: F = 0:15	ME/CFS = 48.3 ± 12.2 HC = 47.9 ± 12.1	Cross-sectional study	3.0T; T1W imaging MPRAGE	fMRI (ASL): resting & PASAT task	(1) Increased functional connectivity (FC) between inferior frontal gyrus and the brainstem and several other brain areas (lingual gyrus, cerebellar vermis, cerebellum, and parahippocampal gyrus) from start to end of the task in ME/CFS but decreased in HC. (2) Greater increases in FC between these areas were positively associated with greater fatigue level (i.e., more fatigued toward the end of the task)
Barnden et al. (42)**	ME/CFS (45) M: F = N/A** HC (27) M: F = N/A**	Not available**	Cross-sectional study	3.0T; T2W imaging “SPACE” optimized 3D fast spin-echo	fMRI (EPI): resting & Stroop task	(1) Deficits in the FC between cuneiform nucleus and medulla, and between brainstem and hippocampus and intralaminar thalamus during the task in ME (2) Reduced connectivity in ME/CFS was associated with increased symptom severity
Thapaliya et al. (43)**	ME/CFS (45) M: F = 12:33** HC (27) M: F = 9:18**	ME/CFS = 47.1 ± 11.7** HC = 43.1 ± 13.7**	Cross-sectional; T1W/T2W ratio assessment	3.0T; T1W imaging MPRAGE; T2W imaging “SPACE” optimized 3D fast spin-echo	None	(1) Increased T1W/T2W ratio (indicative of higher levels of myelin and/or iron) in 16 WM regions (i.e., left and right corticospinal tract, left medial lemniscus, right anterior and left and right posterior limbs of internal capsule, left and right anterior, superior, and posterior corona radiata, left cingulum, forceps minor, sensorimotor, and right inferior longitudinal fasciculus) in ME/CFS compared to HC (2) Increased T1W/T2W ratio in 4 subcortical GM regions (i.e., left and right putamen and pallidum) in ME/CFS compared to HC (3) Voxel-based analysis detected 7 significant clusters with T1W/T2W greater in ME/CFS [globus pallidus, paracentral lobule, posterior corona radiata, left precuneus, putamen, cerebrospinal tract (pons) and medial lemniscus (pons)], consistent with the region findings in 1) (4) Clinical and functional measures (i.e., heart rate variability, SF36 physical, respiratory rate, Stroop Effect) correlated with abnormal T1W/T2W ratio in several brain regions (e.g., cingulate and middle temporal gyri, cerebral WM) in ME/CFS (5) No ME/CFS regions had decreased T1W/T2W ratio compared to HC.
Manca et al. (44)	ME/CFS (6) M: F = 0:6 HC (10) M: F = 0:10	ME/CFS = 42.8 ± 5.7 HC = 39.3 ± 8.7	Repeated measure of MRI; pilot study with 2 scans several days apart	3.0T; T1W imaging MPRAGE; T2-FLAIR	fMRI (EPI): resting	(1) No regional or global group mean differences in GM, WM, or WM hyperintensity volumes (2) Altered baseline FC between default mode network seeds (right inferior parietal lobules and posterior cingulate cortex) and frontal areas (left and right superior frontal gyrus and left precentral gyrus) in ME/CFS compared to HC (3) Greater baseline FC between salience network seeds (anterior cingulate, right and left insula) and left superior temporal gyrus; and between both insulae and the medulla and cerebellar tonsil in ME/CFS compared to HC (4) Post cognitive exertion, increased FC between right insula and several brain areas (orbito-frontal cortex, thalamus, hypothalamus, and basal ganglia) in ME/CFS compared to HC, with increased fatigue negatively associated with decreased FC between right insula and right inferior temporo-occipital areas; and increased pain associated with altered FC between right insula and several frontal areas (e.g., superior temporal gyrus, inferior frontal gyrus, frontal pole) in ME/CFS.
Addiego et al. (45)	ME/CFS (38) M: F = 11:27 GWI (90) M: F = 70:20 HC (34) M: F = 22:12	ME/CFS = 47.7 ± 13.0 GWI = 47.5 ± 7.5 HC = 43.7 ± 16.6	Cross-sectional study; volumetric analysis	3.0T; T1W imaging MPRAGE	None	(1) Decreased adjusted volume for left putamen, right caudate and left cerebellum WM in ME/CFS compared to HC (2) In ME/CFS, brainstem volume correlated with several brain region volumes (left and right ventral diencephalon, right pallidum, and left cerebellum WM) (3) In HC, brainstem volume correlated with several other brain region volumes (right hippocampus and caudate, left and right diencephalon, thalamus, cerebellum cortex, and pallidum)
Thapaliya et al. (46)**	ME/CFS-F (25) M: F = 5:20** ME/CFS-ICC (18) M: F = 6:12** HC (26) M: F = 9:17**	ME/CFS-F = 49.8 ± 12.2** ME/CFS-ICC = 43.3 ± 10.7** HC = 43.1 ± 13.7**	Cross-sectional study	3.0T; DTI	None	(1) Decreased DTI parameters [i.e., axial diffusivity, mean diffusivity, mode of anisotropy, transverse eigenvalue (λ2)] in several brain regions (*i.e*., midbrain/pons, superior longitudinal fasciculus) in ME-ICC compared to HC (2) Increased DTI parameter (λ3) in medulla in ME-ICC compared to HC (3) No group difference in DTI parameters in any brain regions between ME-F and HC (4) Opposite correlation patterns between ME-ICC and HC in DTI parameters (i.e., fractional anisotropy, axial diffusivity, mean diffusivity, radial diffusivity, mean of anisotropy) of several brain regions (e.g., parahippocampal gyrus, corpus callosum, hippocampus, posterior cingulate, external capsule) vs. clinical measures (e.g., information processing score, respiratory rate, SF36 physical, sleep disturbance score)

* *Indicating same cohort and data is available in another Table entry (1)*.

** *Indicating that these entries mostly applied the same cohort as Table entry (8). M: F, males: females; MRI, magnetic resonance imaging; SD, standard deviation; ME/CFS, myalgic encephalomyelitis/chronic fatigue syndrome; M, male; F, female; HC, healthy control; T1W, T1-weighted; T2W, T2-weighted; GRE, gradient echo; GM, gray matter; WM, white matter; PP, pulse pressure; BP, blood pressure; HR, heart rate; MPRAGE, magnetization prepared rapid gradient echo; TIV, total intracranial volume; fMRI, functional magnetic resonance imaging; ASL, arterial spin labeling; PASAT, Paced Auditory Serial Addition Test; FC, functional connectivity; SPACE, Sampling Perfection with Application optimized Contrast; EPI, echo-planar imaging; T2-FLAIR, T2-weighted Fluid-Attenuated Inversion Recovery; GWI, Gulf War Illness; ME/CFS-F, ME/CFS Fukada criteria; ME/CFS-ICC, ME/CFS International Consensus Criteria*.

### Synthesis of Results

The studies were summarized for main findings. Data was grouped and presented in the context of structural or functional brain changes. For each study, participant characteristics including mean age, MRI techniques including the sequence, study design, publication year, and main findings concerning the brainstem-specific and non-brainstem were provided. Studies included in other reviews and meta-analyses on ME/CFS research that met the criteria for the present review (i.e., MRI research on the brainstem in ME/CFS) were included.

## Results

**Figure 2:**
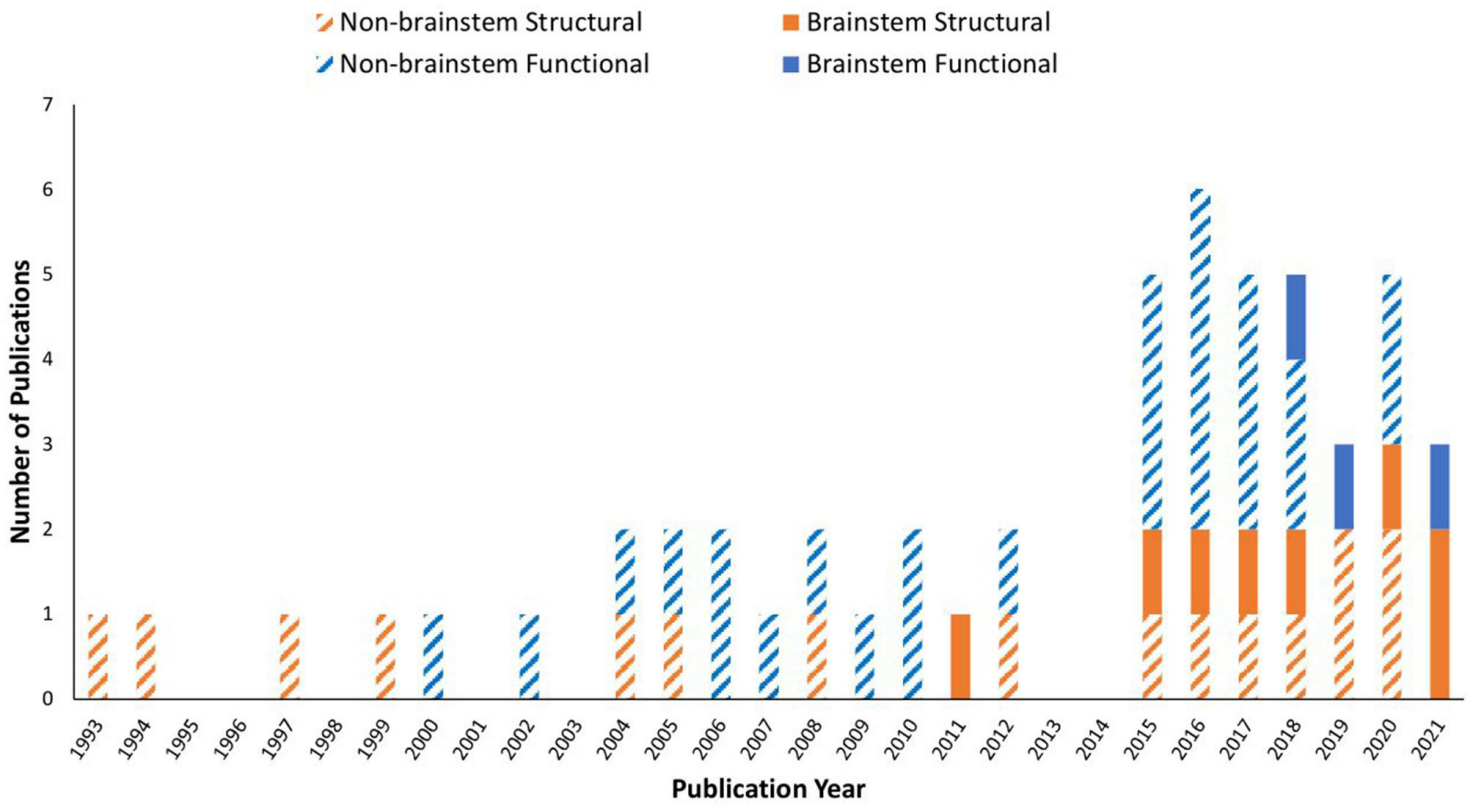
Publication trend in brain MRI research on myalgic encephalomyelitis/chronic fatigue syndrome (ME/CFS) from 1993 to 2021. A MEDLINE and PubMed search using the keywords described yielded a total of 53 MRI original research papers. Studies investigated the brainstem are shown in filled colors, while those excluded the brainstem are in striped colors: orange: structural MRI only; blue: functional MRI involved.

Data showed that ME/CFS has been a subject of MRI studies for decades. Even so, it was not until the past decade when the brainstem emerged in the research line applying MRI, especially in terms of brainstem function (Table 1 and Figure 2). The fMRI studies were published in 2018, 2019, and 2021, using perfusion or BOLD based techniques. Seven of the articles (while some of them used the same dataset) enrolled both adult sexes/genders; two enrolled only adult women; one included Gulf War Illness participants together with ME/CFS and controls; two missed presenting the sex/gender information. The ME/CFS participants in the 11 studies aged from 31.7 ± 8.8 to 49.8 ± 12.2 compared to 32.8–48.4 for healthy controls (HC). The studies all used structural MRI, chiefly T1W imaging for examining the anatomy and the co-registration of the functional images upon structural ones. Six studies also acquired T2W imaging and one acquired T2-weighted Fluid-Attenuated Inversion Recovery (T2-FLAIR) to more effectively investigate pathological changes, and one study pioneered the use of DTI in understanding the brainstem. Most studies performed a one-time MRI scan, except for one that had two scans separated by several days (Table 1). It is noted that the MRI studies investigating the brainstem's role in ME/CFS were conducted by a limited number of research groups (Table 1).

### Structural Brain Changes in ME/CFS

Barnden et al. (36) reported changes in several brain structures (brainstem, prefrontal WM, and hypothalamus) in ME/CFS using a sample consisting of 25 ME/CFS patients and 25 HC. By regressing volumetric brain MRI data and ME/CFS clinical scores, the authors identified a negative correlation between midbrain WM volume (explained as a loss/shrinkage of glial cells) and the duration of fatigue. By comparing regressions between brain MRI measures and physiological measures across the two groups, the authors reported group differences suggesting abnormalities of the brain areas involved in ME/CFS. For example, the absolute GM volume of the brainstem and the seated pulse pressure (PP) were positively correlated in ME/CFS, but not in HC. The correlation between WM volume of the basal pons and the seated diastolic blood pressure (BP) was positive in ME/CFS, but negative in HC. The seated PP was also correlated with the T1W signal level (suggestive of myelination) in the brainstem and cerebellum, negatively in ME/CFS and positively in HC. Regressions between the asleep heart rate and the WM volume of the prefrontal cortex, hypothalamus, and cerebellar vermis also differed between the two groups (36). The study found no group mean differences in T1W or T2W signal levels, or WM or GM volumes, in the whole brain or in any specific brain regions.

Later, the research group published additional results using the same dataset (37). In addition to assuring that the midbrain WM volume was smaller in ME/CFS patients with longer periods of illness onset, the authors further reported an increasing T1W signal level (suggestive of increased myelination) in the ventrolateral thalamus, internal capsule, and prefrontal WM with increased disease severity. Other findings reported by this study included increased T2W signal level (suggestive of decreased regional blood volume) in the right middle temporal WM in ME/CFS, compared to HC.

Again, analyzing the same dataset, the research group further focused on understanding the involvement of the vasomotor centers in the brainstem and the hypothalamus in autonomic functional changes in ME/CFS (38). They reported abnormal correlations between multiple MRI measures in focal brainstem structures and physiological measures in ME/CFS compared to HC (e.g., medulla T2W signal level; pons WM volume; hypothalamus T1W signal level and WM volume; cuneiform nucleus of the reticular formation and ventral tegmental T1W signal level; erect PP and BP, reclined systolic BP and heart rate). The authors also found no group differences in these structures such as T1W or T2W signal level, or GM or WM volumes. The study found opposite correlation patterns involving additional brain structures (limbic nuclei, and prefrontal WM) with physiological measures for ME/CFS and HC.

Finkelmeyer et al. investigated brainstem abnormalities in ME, focusing on detecting a regional difference in the WM or GM volume (39). The study reported reduced WM volume in the midbrain and pons in ME/CFS compared to HC. A reduced WM volume in the internal capsule, external capsule, prefrontal lobe, inferior frontal lobe, and temporal lobe was also reported for ME/CFS. Surprisingly, the study reported higher adjusted GM volumes in the temporal, frontal, and occipital lobes in ME/CFS patients compared to HC but a possible explanation of the group difference was not found. Other findings included reduced total intracranial volume (TIV), increased TIV-adjusted global GM volume, and decreased TIV-adjusted global WM volume, and negative associations between symptom severity and the TIV, TIV-unadjusted GM, and cerebrospinal fluid volume in ME/CFS.

Barnden et al. (40) employed a larger sample (compared to their earlier publications) of 43 ME/CFS patients and 27 HC. The authors reported decreased T1W signal levels (suggestive of decreased myelination) in the brainstem (ventral tegmental, pontine nuclei, medulla regions) and increased T1W signal levels in cortical WM regions (i.e., premotor and sensorimotor cortices) in ME/CFS compared to HC (as in the internal capsule as they reported in the 2015 paper). Negative correlations in T1W signal levels between the brainstem (low myelination) and sensorimotor cortex (high myelination) were found in both groups.

Thapaliya et al. applied a novel T1W/T2W ratio (a higher ratio is indicative of higher levels of myelin and/or iron), to investigate differences in the levels of myelin and/or iron using 45 ME/CFS and 27 HC participants (43). The study reported increased ratio in ME/CFS compared to HC in 16 WM regions (i.e., brainstem regions-of-interest like the corticospinal and medial lemniscus tracts), projection tracts including the internal capsule, corona radiata, and others (cingulum, forceps minor, sensorimotor, and inferior longitudinal fasciculus). Also reported was the increased ratio in all four subcortical GM regions studied (i.e., the left and right putamen and pallidum) in ME/CFS compared to HC. The abnormality was correlated with clinical and functional measures. A lower ratio was not found in any brain regions in ME/CFS compared to HC, reflecting lack of increased demyelination.

Recently, Addiego et al. (45) investigated the volume of brain regions across three participant groups: 38 ME/CFS, 90 Gulf War Illness, and 34 HC participants. The study showed decreased volume for the putamen, caudate and cerebellum WM in ME/CFS compared to HC, adjusted for intracranial volume and age. The brainstem volume was correlated with the volume of several other brain regions including ventral diencephalon, pallidum, cerebellum WM in ME/CFS. The correlations were in contrast to those observed in HC in different brain regions (e.g., hippocampus, caudate, diencephalon, thalamus, cerebellum cortex).

Most recently, Thapaliya et al. (46) conducted the only DTI study so far, investigating the brainstem in ME/CFS. The authors examined 25 ME/CFS-F (a subgroup meeting the Fukuda criteria), 18 ME/CFS-ICC (a subgroup meeting the International Consensus Criteria) and 26 HC participants. They reported decreased transverse diffusivity and fractional anisotropy in several brain regions including midbrain/pons and superior longitudinal fasciculus in ME/CFS-ICC compared to HC. In addition, this patient subgroup showed correlations between the DTI and clinical measures in several brain regions (e.g., external capsule, corpus callosum). No group differences were seen in DTI parameters in any brain regions between ME/CFS-F and HC.

### Functional Brain Changes in ME/CFS

Boissoneault et al. published the first fMRI study on ME/CFS examining the brainstem in 2018 in 19 ME/CFS and 15 HC participants (Table 1). The authors examined changes in FC during the Paced Auditory Serial Addition Test (PASAT, a fatiguing cognitive task). Participants were presented with numbers sequentially and were asked to determine whether the sum equaled 13, while self-reporting their fatigue level. The study showed progressively increased FC between the inferior frontal gyrus (IFG) and the brainstem and several other brain areas (lingual gyrus, cerebellar vermis, cerebellum, parahippocampal gyrus) during the task in ME/CFS patients, in contrast to a decrease in FC involving the IFG in HC (41). Such increase in FC in ME/CFS was associated with higher fatigue level toward the end of the task.

Another fMRI study of the brainstem in ME/CFS confirmed an impairment in brainstem nerve conduction in ME/CFS (42). The study assessed FC within, and from, the brainstem using a sample of 45 ME/CFS patients and 27 HC. Participants were asked to complete a version of the Stroop task (for word meaning and color matching). The study reported connectivity deficits between the cuneiform nucleus and medulla, and from the brainstem to both the hippocampus and intralaminar thalamus in ME/CFS. The reduced connectivity was associated with an increased level of symptom severity.

Manca et al. (44) investigated FC changes associated with post-exertional malaise in the default mode network and the salience network (key for autonomic function), in six ME/CFS and 10 HC participants (44). Baseline FC data were collected, and group differences were examined, following which a series of cognitively fatiguing tasks were administrated (Stroop task, Trail making test, Category Fluency Task, Letter Fluency task, PASAT, N-back task) to elicit post-exertional malaise. Participants had another MRI scan several days later (at the onset of post-exertional malaise for ME/CFS patients). The study showed differential baseline FC in ME/CFS and HC (e.g., greater between the right inferior parietal lobules and both the posterior cingulate cortex and the right superior frontal gyrus; lower between the posterior cingulate cortex and both the left superior frontal gyrus and left precentral gyrus in ME/CFS). Greater baseline FC in ME/CFS was also found between the salience network (seeds in the anterior cingulate and the right and left insula) and the left superior temporal gyrus; and between both insulae and the medulla and cerebellar tonsil. At the second scan time, increased FC between the right insula (and the orbito-frontal cortex, thalamus, hypothalamus, and basal ganglia) and decreased FC between the right insula and right inferior temporo-occipital were found in ME/CFS, companied by increased level of fatigue and pain.

## Discussion

### Summary and Interpretation of the Findings

We conducted a literature review to summarize and evaluate MRI research findings to better understand the role of the brainstem in ME/CFS. The brainstem has only been subject to focused MRI investigations for a decade, with the functional studies seen only since 2018 (Figure 2). Even so, the research published so far has demonstrated the critical involvement of the brainstem in ME/CFS associated deficits (Table 2).

**Table 2:** Number of studying reporting structural and functional brain MRI changes in relation to myalgic encephalomyelitis/chronic fatigue syndrome (ME/CFS).

**Findings**	**Number of studies**					
	**Structural**					**Functional**
	**Myelination**	**White matter volume**	**Gray matter volume**	**Total intracranial volume**	**Circulation**	**Functional connectivity**
No regional or global group mean differences	3	3	2	0	2	*
Abnormal correlation with physiological measures	4	2	1	0	1	0
Correlation with symptom severity	1	0	1	1	0	3
Group mean differences	0	0	0	1	0	3
Regional group mean differences	2	2	1	0	1	*
Correlation with disease duration	0	2	0	0	0	0
Evidence of cortical compensation	3	0	0	0	0	0

* *Indicating this brain MRI change has not been studied/reported*.

The studies pointed to a reduced WM volume (36–39), impairments in myelination (36, 38, 40, 43, 46), reduced conduction (37, 38, 40, 46), abnormal FC linking the brainstem and other brain regions (41, 42, 44), and compensatory brain changes (37, 40). The research findings also demonstrated a linkage between brainstem abnormalities and other brainstem structures, highlighting myelination impairment and dysregulation underlying the disease (Table 2 and Figure 2). The reports of midbrain WM volume loss, brainstem myelination impairments, and connectivity deficits within the brainstem also indicated brainstem nerve conduction impairment in ME/CFS, even when the brainstem nuclei themselves remained unaffected. Meanwhile, the increased myelination of multiple brain regions (ventrolateral thalamus, internal capsule, and prefrontal, premotor and sensorimotor cortices) showed a compensatory response in maintaining nerve conduction (e.g., increased myelination of tract segments rostral to the brainstem). Also in support of the regulatory hypothesis, the region based approach in Thapaliya et al. (43) detected increased T1W/T2W ratio in the internal capsule, coronal radiata, cingulum forceps minor, sensory motor, and inferior longitudinal fasciculus, while the voxel based analysis, which does not require a-priori region selection and tests the whole brain, detected increased T1W/T2W ratio in the globus pallidus, paracentral lobule, posterior corona radiata, left precuneus, putamen, cerebrospinal tract (pons) and medial lemniscus (pons).

**Figure 3:**
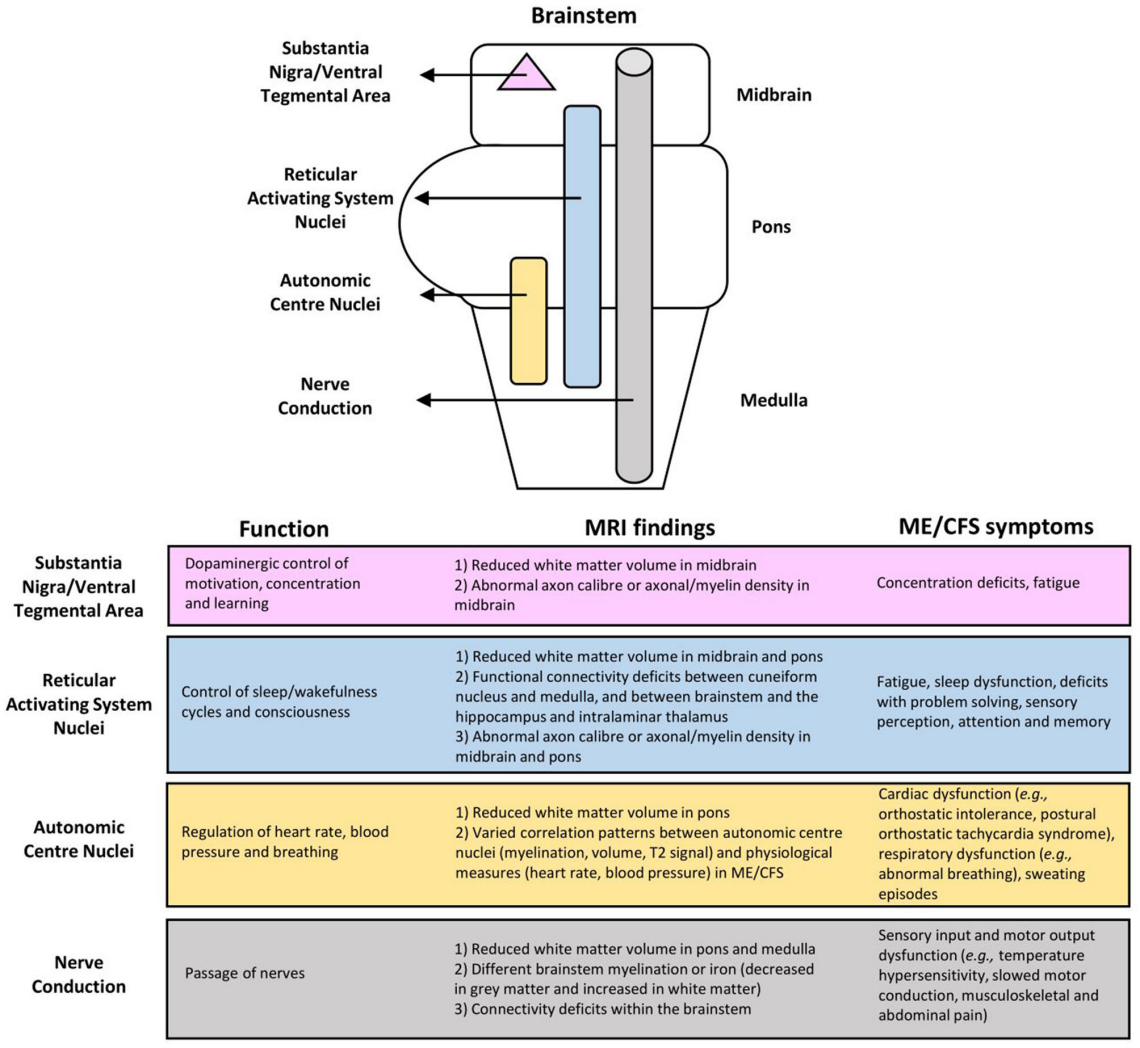
Associations of myalgic encephalomyelitis/chronic fatigue syndrome (ME/CFS) symptoms with brainstem dysfunction based on MRI findings.

As summarized in Figure 3, the MRI brain changes in ME/CFS may reflect dysfunctions of the brainstem in several key aspects, including autonomic control and regulation, and afferent and efferent conduction. Such dysfunctions in the brainstem can have consequences on ME/CFS, relating to symptoms such as fatigue, physiological dysregulation, and even, most likely secondary, cognitive decline (7, 8).

The limited number of existing fMRI studies demonstrated clear abnormalities in the neural network connectivity between several brain areas thought to be involved in ME/CFS symptomology (Figure 3). Abnormalities of the RAS nuclei of the brainstem, which are crucial for the coherence of thalamo-cortical oscillations, are suggested to result in the dysfunction of problem solving, sensory perception, attention, and memory in ME/CFS (42). Abnormalities within the default mode network are suggested to play a role in post-exertional malaise (44), and spatial sensing and coordination difficulties (33), while those within the salience network possibly reflect an increased challenge of salience processing with cognitive performance, interoceptive processing, and visceromotor control related to fatigue and pain perception in ME/CFS (44). Abnormalities from the brainstem to the prefrontal lobe [e.g., the IFG, the known executive control centre; (47)] could lead to executive dysfunctions, while abnormalities between the brainstem and other brain regions (i.e., lingual gyrus, cerebellar vermis, cerebellum, parahippocampal gyrus) are suggestive of a failure of compensatory mechanisms for fatigue (41).

Also importantly, these MRI brain changes were associated with, and supported by, a wide range of clinical assessments, including the vital signs (36, 38, 43, 46), sleep parameters (36, 38, 46), disease duration (36, 37) and severity levels (37, 39, 41, 42, 44).

### Challenges of the Research Field

Our study has identified several challenges of the field. Notably, controversies exist in identifying mean group differences in regional or global T1W and T2W signal levels, or GM and WM volumes in ME/CFS compared to controls. The initial conventional 1.5 Tesla MRI studies failed to detect such a change using relatively small samples (36, 38). More recent studies (37, 39, 40, 43, 45) were able to detect such differences with increased sample sizes, especially at 3.0 T high-field MRI (Table 1). Notably, most MRI on ME/CFS studies thus far applied a relatively small sample size of ME/CFS patient participants, ranging between 6 and 45. Finkelmeyer et al. (39) attributed the lack of regional differences in WM and GM volumes to small sample sizes used, even though the study included 42 patients. The present study informs future research to pay a closer attention to enforcing the effect size to allow adequate statistical power in detecting a targeted difference. Also, several studies under review failed to provide the basic demographics of the study sample. Considering that ME/CFS is a multi-system disease with heterogeneous symptom expression among individuals, a thorough description of the sample will allow appropriate result interpretation and the evaluation of the generalizability of the study finding.

Despite the widely available MRI technologies as applied in studying other clinical conditions (48), they have yet to be sufficiently employed in better understanding ME/CFS. For example, structural scans have only involved T1W and T2W sequences in investigating the brainstem, save for the most recent pioneer DTI work (46). Future research using different MRI techniques, including DTI and other advanced methods may help detect WM changes more sensitively. Furthermore, MRS studies can be invaluable to understanding brainstem metabolites, as widespread brain abnormalities have been reported as suggestive of neuroinflammation in correlation with ME/CFS symptom severity (22). It is anticipated that multimodal MRI studies combining various MRI methods investigating the brainstem can be beneficial in providing confirmatory evidence. Certainly, these undertakings can require vigilant experimental design and implementation, given that the brainstem is deeply located in the brain and relatively difficult to shim and image (21).

Also importantly, to date, the MRI on brainstem publications have largely shown a group-wise comparison of the mean values and the regression patterns, and adopted cross-sectional design with single-time MRI. Studies with longitudinal follow-ups will be needed to understand the progress of the brain deficits and the consequences regarding disease expression. Similarly, clinical trials examining the effect of standard care and novel interventions have yet to become available. Clinical trials are important especially given the consideration that ME/CFS has been recently suggested to be one of the long-term consequences of COVID-19 and has been estimated to double in its prevalence in 1 year in the United States (34), and likely also in other countries.

Over the past decades, MRI on ME/CFS has drawn an increasingly greater attention, as demonstrated by the increase in the number of studies, specifically functional ones (Figure 2). This has reflected, and helped with, the growing body of recognition of ME/CFS as a medical condition, which has become the topic of several recent reviews. While structural and functional abnormalities in ME/CFS are typically found, inconsistencies exist among individual studies (30, 49). Impairments in the autonomic nervous system in ME/CFS are also detected by other imaging methods including positron emission tomography and electroencephalography (50). Unfortunately, most MRI on ME/CFS studies till now have not targeted the role of the brainstem (Figure 1).

### Limitations of the Study

Our study has several limitations. First, we applied only the MEDLINE database. Even though it is well-established and provides a comprehensive coverage of peer-reviewed biomedical articles in biomedicine of recognized quality, some abstracts, conference reports, etc., indexed only on less prominent resources might have been excluded. Our findings have also been limited to reports published in English and those targeting the brainstem using structural or functional MRI. Reports with other focuses outside of the scope of the present research may warrant separate reviews (Figure 3). As demonstrated by the present review, MRI studies investigating the brainstem's role in ME/CFS are emerging quickly, calling for future attentive actions of MRI researchers.

### Involvement of the Brainstem

Our research has made a helpful contribution to the literature. The study integrated the MRI findings on the role of the brainstem in ME/CFS and revealed brainstem abnormalities in relation to various symptoms that present heterogeneously in patients. The review has also resulted in a model for better understanding the role of the brainstem dysfunction in ME/CFS, showing changes within the brainstem, and linking with other brain regions, as detailed below (Figure 3).

First, the abnormal connectivity between RAS nuclei possibly lessened the cortical oscillatory coherence as they control the thalamocortical state between sleeping and wakefulness, which can consequently affect consciousness and cognitive function (19, 20). Also, given the distribution of the RAS nuclei throughout the brainstem (11), their functions may be affected by the reduced WM volume and functional connectivity, and the suggested abnormal axon caliber and axonal/myelin density in the midbrain and pons (46). RAS dysfunction may lead to fatigue, impaired attention, short term memory, cognitive function, and sleep quality, all of which have been seen in the ME/CFS population (7, 8).

Also vital for autonomic functions, the brainstem plays a role in the regulation of heart rate, blood pressure, and respiration (15, 16). The reduced WM volume in the pons and the varied correlation pattern of the autonomic centre nuclei on physiological measures reflect autonomic centre dysfunction in ME/CFS, such as cardiac (e.g., postural orthostatic tachycardia syndrome, orthostatic intolerance, arrhythmia); respiratory (e.g., abnormal breathing); and other dysfunctions including sweating episodes (7, 8).

Impairments also include the crucial passage of nerves (i.e., conduit function) in both afferent and efferent directions. Reduced WM volume in the pons and medulla, different brainstem myelination or iron (decreased in GM and increased in WM), and connectivity deficits within the brainstem in ME/CFS are indicative of conduit dysfunction. Symptoms may arise directly from the affected nerves and/or indirectly from cortical myelination as a compensatory response. Related symptoms may include sensory input and motor output dysfunctions. Meanwhile, patients with excessive myelination of tracts to the primary somatosensory cortex may have increased sensory awareness, which can manifest into symptoms such as increased touch and pain perception (8, 51, 52), musculoskeletal and abdominal pain (8, 53), and intolerance to extreme temperatures (8). Alternatively, the prolonged motor conduction velocity in ME/CFS (54), which is indicative of motor disturbances (8) may be attributed to insufficient myelination (i.e., under compensation) of tracts from the motor cortex.

Moreover, the midbrain uniquely contains the substantia nigra and ventral tegmental area for dopaminergic control of motivation, concentration, and learning (17, 18). Dopaminergic dysfunction in ME/CFS has been suggested but is yet to be tested by MRI. Specifically, it has been shown that methylphenidate, a dopamine reuptake inhibitor, decreases the levels of fatigue and disrupted concentration in adults with ME/CFS (55). Functional impairment of the putamen, which is innervated by, and is a part of the same functional network as the substantia nigra and ventral tegmental area, has also been reported in ME/CFS (24), suggesting a topic for future investigation.

The review is in favor of the hypothesis that ME/CFS involves brainstem specific astrocyte dysfunctions, contributing to impaired brainstem cerebrovascular autoregulation and reduced blood flow (36, 56). Oxidative stress appears to induce ME/CFS symptoms, associated with reduced blood flow and neuroinflammation. An infection (the most frequently reported trigger for ME/CFS onset) has been linked to peroxynitrite production, a proinflammatory oxygen/nitrogen species (57, 58), triggering neuroinflammation. This can lead to the production of isoprostanes and cause vasoconstriction when the level of antioxidants is insufficient (59). Oxidative stress and increased isoprostanes in ME/CFS have been correlated with clinical symptoms (60). Reduced brain blood flow is common in ME/CFS patients, accompanied by lowered circulation and nutrient/waste exchange (61–65).

Taken together, the present review highlights the brainstem as a potential brain centre that may have a key role in the physiological defects in ME/CFS and recommends future MRI on ME/CFS studies to make the brainstem a specific target. Given the close association between the brainstem function impairments and the complex clinical expression of the disease, MRI, with its relatively high spatial and temporal resolutions for non-invasive *in vivo* applications, holds promise to uncover the mechanisms of the disease, and in turn enlightens effective strategies for improved patient care. This review contributes to the research line by bringing the existing studies together and integrating them while highlighting the potential for more. As the big questions around ME/CFS remain unanswered (e.g., why does an initial viral event result in a debilitating ongoing life-long disease in some people, why does it not resolve like a typical infectious illness, and why are ME/CFS patients subject to frequent relapses), highlighting of the brainstem as a specific target is meaningful from both research and clinical practice perspectives.

## Conclusion

This study targets a better understanding of the involvement of the brainstem in ME/CFS using MRI. Data demonstrated that structural and functional deficits of the brainstem are associated with other brain changes and linked to clinical expression. The study suggested a model summarizing the possible brainstem role in connecting various functional brain centres. The paper draws increased attention to brainstem research in ME/CFS using multi-modality MRI, calling for improved experimental design, and increased sample size and follow-up duration. Targeting the brainstem abnormalities in relation to the heterogeneous symptoms has implications for uncovering ME/CFS mechanisms and thus improving management and patient care.

## Author Contributions

TN and LXZ conducted the literature search, review and evaluation, summarized and presented the results, and drafted the initial version of the manuscript. HG provided consultations on MRI technologies and evaluations and the diagnostic medical imaging, and reviewed the result and data presentation. LN enabled the funding support, provided medical consultations about the evaluations and treatments of the medical condition, reviewed the result presentation, and edited the manuscript. XS enabled funding support, conceptualized and supervised the study, reviewed the literature evaluation and result presentation, and co-drafted the first version of the manuscript. All authors participated in the result interpretation and the manuscript revisions.

## Funding

This study received research grant support from the British Columbia Strategy for Patient Oriented Research Unit (FHG2020-014; FHG2020-005) and the Surrey Hospitals Foundation (FHG2017-001). Additional support for training was from the Natural Sciences and Engineering Research Council of Canada (USRA-507466) and the BioTalent Canada (SWPP-FH2020).

## Conflict of Interest

The authors declare that the research was conducted in the absence of any commercial or financial relationships that could be construed as a potential conflict of interest.

## Publisher's Note

All claims expressed in this article are solely those of the authors and do not necessarily represent those of their affiliated organizations, or those of the publisher, the editors and the reviewers. Any product that may be evaluated in this article, or claim that may be made by its manufacturer, is not guaranteed or endorsed by the publisher.

## Abbreviations

ASL: arterial spin labeling
BOLD: blood oxygenation level dependent
DTI: diffusion tensor imaging
EPI: echo-planar imaging
FC: functional connectivity
fMRI: functional magnetic resonance imaging
GM: gray matter
HC: healthy controls
IFG: inferior frontal gyrus
ME/CFS: myalgic encephalomyelitis/chronic fatigue syndrome
MRI: magnetic resonance imaging
MRS: magnetic resonance spectroscopy
PASAT: paced auditory serial addition test
PP: pulse pressure
RAS: reticular activating system
TIV: total intracranial volume
T1W: T1-weighted
T2W: T2-weighted
T2-FLAIR: T2-weighted fluid-attenuated inversion recovery
WM: white matter.
